# Effect of mirabegron on cognitive function in elderly patients with overactive bladder: MoCA results from a phase 4 randomized, placebo-controlled study (PILLAR)

**DOI:** 10.1186/s12877-020-1474-7

**Published:** 2020-03-18

**Authors:** Tomas L. Griebling, Noll L. Campbell, Jeffrey Mangel, David Staskin, Sender Herschorn, Dina Elsouda, Carol R. Schermer

**Affiliations:** 1 Department of Urology and The Landon Center on Aging, University of Kansas School of Medicine, Kansas City, KS USA; 2 College of Pharmacy, Purdue University, Lafayette, IN USA; 3 Center for Aging Research, Indiana University, Indianapolis, IN USA; 4 Division of Urogynecology and Pelvic Reconstructive Surgery, MetroHealth Medical Center, Cleveland, OH USA; 5 Division of Urology, St Elizabeth’s Medical Center, Boston, MA USA; 6 Division of Urology, Sunnybrook Health Sciences Centre, University of Toronto, Toronto, Ontario Canada; 7 Medical Affairs, Astellas Pharma Global Development, Inc., Northbrook, IL USA

**Keywords:** Aged, Clinical trial, Phase 4, Cognition, Lower urinary tract symptoms, Urinary bladder, Overactive, Elderly, Geriatric

## Abstract

**Background:**

Antimuscarinics are often used for treatment of overactive bladder (OAB), but exposure to medications such as antimuscarinics that have anticholinergic properties has been linked to adverse cognitive effects. A phase 4 placebo-controlled study (PILLAR; NCT02216214) described the efficacy and safety of mirabegron, a β_3_-adrenoreceptor agonist, for treatment of wet OAB in patients aged ≥65 years. This pre-planned analysis aimed to measure differences in cognitive function between mirabegron and placebo, using a rapid screening instrument for mild cognitive impairment: the Montreal Cognitive Assessment (MoCA).

**Methods:**

Outpatients aged ≥65 years with wet OAB were randomized 1:1 to mirabegron or placebo, stratified by age (<75/≥75 years). There were no exclusion criteria regarding cognitive status. Patients randomized to mirabegron initially received 25 mg/day with an optional increase to 50 mg/day after week 4/8 based on patient/investigator discretion. The MoCA was administered at baseline and end of treatment (EoT, week 12). The study protocol was Independent Ethics Committee/Institutional Review Board-approved.

**Results:**

Of the 887 randomized patients who received ≥1 dose of study drug, 72.3% were female, 79.5% were white, and 28.1% were aged ≥75 years. All patients had ≥1 comorbidity and 94.3% were receiving ≥1 concomitant medication. One third of patients had a history of psychiatric disorders, the most common being depression (17.2%), insomnia (15.7%), and anxiety (11.4%). Baseline mean (standard error, SE) MoCA total scores were 26.9 (0.1) and 26.8 (0.1) in the mirabegron and placebo groups, respectively. Among patients with MoCA data available at baseline/EoT, 27.1% (115/425) and 25.8% (106/411) of mirabegron and placebo group patients, respectively, had impaired cognitive function at baseline (MoCA total score <26). There was no statistically significant change in adjusted mean (SE) MoCA total score from baseline to EoT in the mirabegron group (−0.2 [0.1]) or the placebo group (−0.1 [0.1]).

**Conclusions:**

Treatment with mirabegron for 12 weeks did not contribute to drug-related cognitive side effects in patients aged ≥65 years, as measured by the MoCA. Furthermore, the pattern of change in cognition over time in an older OAB trial population does not appear to differ from that of subjects receiving placebo.

**Trial registration:**

NCT02216214 (prospectively registered August 13, 2014).

## Background

Overactive bladder (OAB) is a symptom complex of storage lower urinary tract symptoms (LUTS) characterized by the presence of urgency. It affects all ages, but is increasingly prevalent as age increases [1–4]. According to the National Overactive Bladder Evaluation (NOBLE) study, prevalence of OAB in those ≥65 years is around 30%, twice as prevalent as in those ≤45 years [2]. By 2025, it is estimated that there will be 52 million adults in the USA with LUTS [5]. The older patient population with OAB has high levels of concomitant medication use and comorbidity, is more likely to experience falls and fractures, and is at increased risk of impairment of activities of daily living [6–8]. However, frail elderly patients with multiple comorbidities are often excluded from clinical trials [9–11].

Antimuscarinics are used to treat OAB, however, the risk of anticholinergic adverse events (AEs) such as dry mouth and constipation increases with age [12]. Exposure to medications with anticholinergic properties has also been linked to adverse cognitive effects, in particular in patients ≥65 years [13]. Furthermore, older people are particularly sensitive to anticholinergic effects as a result of significant age-related decrease in cholinergic neurons/receptors in the brain, reduction in hepatic and renal clearance of medications, and increase in blood-brain barrier (BBB) permeability [14]. In addition, the older patient population is likely to be receiving polypharmacy including other drugs with anticholinergic activity (e.g. tricyclic antidepressants, bronchodilators, ACE inhibitors, and antipsychotics), potentially resulting in a problematic anticholinergic burden, with negative effects on cognitive performance [15]. As a result, the Beers criteria lists all antimuscarinic drugs used for the treatment of OAB as potentially inappropriate for first-line treatment in those ≥65 years of age with dementia or cognitive impairment [16].

There are several screening instruments to detect dementia and cognitive impairment, including the Mini-Mental State Examination (MMSE) [17], the Geriatric Mental State Examination [18] and the Montreal Cognitive Assessment (MoCA) [19]. The 30-item MoCA, a brief cognitive screening tool for mild cognitive impairment (MCI), is recognized as a sensitive measure of cognitive function that can capture declines in cognition over repeated administrations. The MoCA is scaled from 1 to 30, with higher MoCA scores indicating better cognitive function; a MoCA score <26 indicates impaired cognitive function [19]. The MoCA includes six cognitive domains, including visuospatial abilities; language; combined attention, concentration, and working memory; executive function; short-term memory recall; and orientation to time and place, although subsequent consensus has emerged that the total score is most meaningful. The MoCA was conceptualized as an improvement over the MMSE, which does not allow for discrimination between those with MCI and elderly adults with no cognitive impairment. The original context for the MoCA was as a clinical tool, serving front-line physicians in making an initial assessment of MCI.

Mirabegron is a β_3_-adrenoreceptor agonist that represents an alternative OAB treatment to antimuscarinics, and potentially has a more favorable benefit-to-risk ratio in older patient populations [20–23]. The PILLAR study compared flexibly-dosed mirabegron versus placebo in community-dwelling elderly patients ≥65 years with OAB and incontinence [24]. Statistically significant mean improvements in bladder diary parameters were observed for mirabegron versus placebo, and safety and tolerability were in line with the mirabegron safety profile. The PILLAR study is the first to use MoCA to detect potential cognitive decline related to OAB treatment and aimed to measure any differences in change from baseline of MoCA scores between mirabegron and placebo.

## Methods

Full methods are described in the PILLAR primary paper [24], but, in brief, PILLAR was a phase 4, multicentre, 12-week study in the US and Canada. Independent Ethics Committee/Institutional Review Board-approved written informed consent was obtained from all participants or their legally authorized representatives. This study adheres to CONSORT guidelines. Community-dwelling patients aged ≥65 years with wet OAB (≥1 incontinence episode and ≥3 urgency episodes during the 3-day diary, plus an average of ≥8 micturitions/24 h), were randomized in a 1:1 fashion to receive mirabegron or placebo. There were no specific exclusion criteria regarding cognitive status, although patients needed to be able to complete the micturition diaries and questionnaires. Patient-reported medical/surgical history was used to identify comorbidities. Patients who were randomized to mirabegron initially received 25 mg/day, with an optional increase to 50 mg/day after week 4/8 based on patient/investigator discretion. The study was designed and powered to detect a difference on incontinence and micturition frequency between combined mirabegron and placebo groups, and was not powered to detect a difference in MoCA scores. Data are displayed as placebo versus total mirabegron and according to treatment group. The safety analysis set (SAF) included all randomized patients receiving at least 1 dose of study medication. AEs were recorded throughout the study until week 16 (4 weeks post end of treatment [EoT]). The MoCA was the sole safety endpoint for which inferential comparison was pre-specified in the PILLAR statistical analysis plan. Stratified rank analysis of covariance with change in score from baseline was the response variable, with treatment group and gender as fixed factors. An additional ANCOVA included treatment, gender, and MoCA score at baseline as covariates. The MoCA was conducted at baseline and EoT, week 12, by clinic staff trained in its administration, in either English or Canadian French. Information on education level was not collected directly; however, clinic staff were instructed to add 1 point for any participants with ≤12 years education, as per the original MoCA scoring instructions [19]. Differences in change from baseline of MoCA scores between mirabegron and placebo were analyzed using a stratified ANCOVA model. As a complement to the pre-specified analyses, a post hoc analysis of relative risk was conducted to determine the relative risk of change in MoCA score by at least 4 points, which is deemed the minimum detectable change (MDC) [25]. Other post hoc analyses were change from baseline to EoT by baseline medical history of depression or antidepressant medication use, or by presence of ≥1 strong anticholinergic concomitant medication.

## Results

### Baseline

Of the 2380 patients screened, 445 were randomized to mirabegron and 443 to placebo; one patient in the placebo group did not receive treatment (Table 1). Of the 887 randomized patients who received ≥1 dose of study drug, overall 72.3% were female, 79.5% were white, and 28.1% were aged ≥75 years. In total, 226 patients received mirabegron 25 mg and only 219/445 patients (49.2%) uptitrated to 50 mg. There were no meaningful differences in demographic characteristics at baseline between the total mirabegron group and the placebo group. All patients had ≥1 comorbidity and 94.3% were receiving ≥1 concomitant medication; those relevant to cognition are shown in Table 2. One third of patients in the SAF had a history of psychiatric disorders, the most common being depression (17.2%), insomnia (15.7%), and anxiety (11.4%). Charlson Comorbidity Index scores (mean ± SD) were low: 2.3 ± 1.2 for both groups (Table 1).

**Table 1:** Baseline characteristics (safety analysis set)

	Placebo (*n* = 442)	Mirabegron Total (*n* = 445)
Female sex, *n* (%)	324 (73.3)	317 (71.2)
Age, mean ± SD	71.9 ± 6.0	71.7 ± 5.5
Age ≥75 years, *n* (%)	124 (28.1)	125 (28.1)
BMI, kg/m^2^, mean ± SD	30.2 ± 6.4	29.7 ± 6.3
Category, *n* (%)		
<25	91 (20.6)	108 (24.3)
≥25–<30	150 (33.9)	157 (35.3)
≥30	201 (45.5)	180 (40.4)
Ethnicity, *n* (%)		
Not Hispanic or Latino	395 (89.4)	401 (90.1)
Hispanic or Latino	43 (9.7)	41 (9.2)
Unknown	4 (0.9)	3 (0.7)
Race, *n* (%)		
White	357 (80.8)	348 (78.2)
Asian	54 (12.2)	59 (13.3)
Black or African American	25 (5.7)	33 (7.4)
Other	6 (1.4)	5 (1.1)
Country, *n* (%)		
United States	389 (88.0)	385 (86.5)
Canada	53 (12.0)	60 (13.5)
Charlson Comorbidity Index score, mean ± SD	2.3 (1.2)	2.3 (1.2)
History of psychiatric disorders		
Depression	72 (16.3)	81 (18.2)
Insomnia	82 (18.6)	57 (12.8)
Anxiety	42 (9.5)	59 (13.3)
Sleep disorder	5 (1.1)	6 (1.3)
Attention deficit/hyperactivity disorder	4 (0.9)	4 (0.9)
Libido decreased	4 (0.9)	4 (0.9)
Bipolar disorder	3 (0.7)	4 (0.9)
Nicotine dependence	5 (1.1)	1 (0.2)
Adjustment disorder with depressed mood	2 (0.5)	1 (0.2)
Initial insomnia	0	2 (0.4)
Persistent depressive disorder	0	2 (0.4)
Stress	0	2 (0.4)
Major depression	1 (0.2)	1 (0.2)
Adjustment disorder	0	1 (0.2)
Alcoholism	0	1 (0.2)
Burnout syndrome	0	1 (0.2)
Depressed mood	0	1 (0.2)
Drug abuse	0	1 (0.2)
Drug dependence	0	1 (0.2)
Emotional disorder	0	1 (0.2)
Mood swings	0	1 (0.2)
Nervousness	0	1 (0.2)
Post-traumatic stress disorder	0	1 (0.2)
Premature ejaculation	0	1 (0.2)
Anxiety disorder	1 (0.2)	0
Claustrophobia	1 (0.2)	0
Obsessive-compulsive disorder	1 (0.2)	0
MoCA total score^a^, *n* (%)		
Category, *n* (%)		
Normal (≥26)	305 (69.3)	310 (70.0)
Mild (18–25)	103 (23.4)	112 (25.3)
Moderate (10–17)	3 (0.7)	3 (0.7)
Severe (<10)	0	0
Missing	29 (6.6)	18 (4.1)

Safety analysis set (SAF): all randomized subjects who received ≥1 dose of study medication

*MoCA* Montreal Cognitive Assessment, *SD* standard deviation

^a^*N* = 440 for placebo and 443 for mirabegron total

**Table 2:** Concomitant non-OAB medications relevant to cognition during double-blind treatment period

*n* (%)	Placebo (*n* = 442)	Mirabegron Total (*n* = 445)
CONCOMITANT ANTIDEPRESSANTS		
SSRIs	54 (12.2)	53 (11.9)
Citalopram	14 (3.2)	9 (2.0)
Sertraline	11 (2.5)	4 (0.9)
Escitalopram oxalate	5 (1.1)	8 (1.8)
Escitalopram	5 (1.1)	7 (1.6)
Fluoxetine	8 (1.8)	4 (0.9)
Citalopram hydrobromide	3 (0.7)	6 (1.3)
Paroxetine	1 (0.2)	6 (1.3)
Sertraline hydrochloride	4 (0.9)	3 (0.7)
Fluoxetine hydrochloride	3 (0.7)	3 (0.7)
Paroxetine hydrochloride	0	3 (0.7)
Other antidepressants	23 (5.2)	31 (7.0)
Trazodone	13 (2.9)	17 (3.8)
Duloxetine	3 (0.7)	4 (0.9)
Duloxetine hydrochloride	4 (0.9)	2 (0.4)
Mirtazapine	2 (0.5)	3 (0.7)
Trazodone hydrochloride	0	3 (0.7)
Venlafaxine hydrochloride	2 (0.5)	1 (0.2)
Desvenlafaxine succinate	0	1 (0.2)
Nefazodone	0	1 (0.2)
Oxitriptan	0	1 (0.2)
Venlafaxine	1 (0.2)	0
Vortioxetine	1 (0.2)	0
Non-selective monoamine reuptake inhibitors	7 (1.6)	9 (2.0)
Amitriptyline	2 (0.5)	7 (1.6)
Doxepin	1 (0.2)	1 (0.2)
Nortriptyline	2 (0.5)	0
Amitriptyline hydrochloride	0	1 (0.2)
Imipramine	1 (0.2)	0
Nortriptyline hydrochloride	1 (0.2)	0
Diazepines, oxazepines, thiazepines and oxepines	3 (0.7)	1 (0.2)
Quetiapine fumarate	3 (0.7)	1 (0.2)
Other antipsychotics	0	2 (0.4)
Aripiprazole	0	1 (0.2)
Risperidone	0	1 (0.2)
CONCOMITANT ANTICHOLINERGICS		
Antihistamines for systemic use	38 (8.6)	41 (9.2)
Hydroxyzine	2 (0.5)	2 (0.4)
Hydroxyzine hydrochloride	1 (0.2)	1 (0.2)
Aminoalkyl ethers	15 (3.4)	16 (3.6)
Diphenhydramine hydrochloride	10 (2.3)	11 (2.5)
Diphenhydramine	4 (0.9)	4 (0.9)
Dimenhydrinate	1 (0.2)	1 (0.2)
Carbinoxamine maleate	0	1 (0.2)
Piperazine derivatives	3 (0.7)	2 (0.4)
Meclozine	2 (0.5)	1 (0.2)
Phenothiazine derivatives	1 (0.2)	1 (0.2)
Promethazine	1 (0.2)	0
Promethazine hydrochloride	0	1 (0.2)
Substituted alkylamines	0	2 (0.4)
Chlorphenamine maleate	0	2 (0.4)
Cough and cold preparations	17 (3.8)	20 (4.5)
Promethazine with codeine	0	1 (0.2)
Carbamic acid esters	5 (1.1)	1 (0.2)
Carisoprodol	2 (0.5)	1 (0.2)
Synthetic anticholinergics, esters with tertiary amine groups	0	2 (0.4)
Dicycloverine hydrochloride	0	2 (0.4)

*OAB* overactive bladder*, SSRIs* selective serotonin reuptake inhibitors

**Fig. 1:**
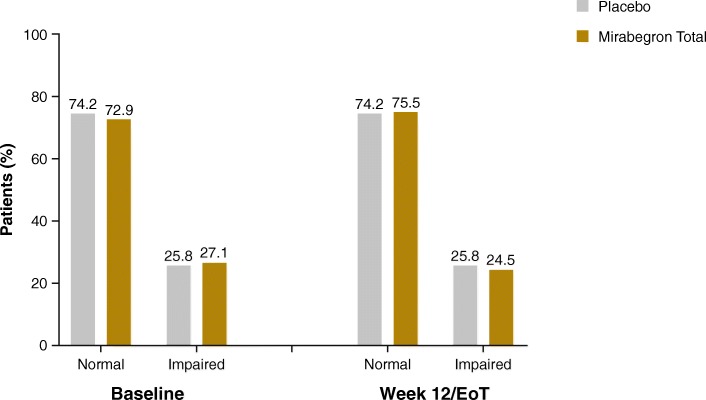
MoCA score at baseline and week 12/EoT. *EoT* end of treatment, *MoCA* Montreal Cognitive Assessment. Impaired cognitive function = MoCA total score <26 [19]

Baseline mean (standard error, SE) MoCA total scores were 26.9 (0.1) and 26.8 (0.1) in the mirabegron and placebo groups, respectively (Table 3). Among patients with MoCA data available at baseline/EoT, 27.1% (115/425) and 25.8% (106/411) of mirabegron and placebo group patients, respectively, had impaired cognitive function (MoCA total score <26) at baseline (Fig. 1).

**Table 3:** Change from baseline to EoT in MoCA test total score (SAF)

	Placebo	Mirabegron Total
Baseline, mean (SE)^a^	26.8 (0.1)	26.9 (0.1)
EoT, mean (SE)^b^	27.0 (0.1)	26.9 (0.1)
Adjusted change from baseline, mean (SE)	−0.1 (0.1)	−0.2 (0.1)
95% two-sided CI	(−0.4, 0.2)	(−0.4, 0.1)
*p*-value		0.471

*CI* confidence interval, *EoT* end of treatment, *MoCA* Montreal Cognitive Assessment, *SAF* safety analysis set, *SE* standard error

^a^*N* = 440 for placebo and 443 for mirabegron total

^b^*N* = 413 for placebo and 427 for mirabegron total

### End of treatment

**Fig. 2:**
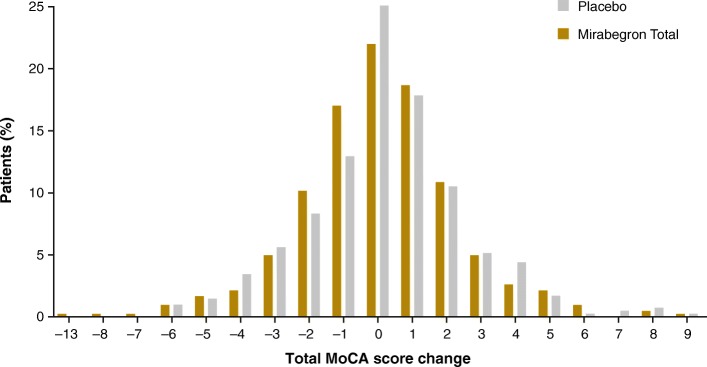
Percentage of patients with indicated MoCA score change at week 12/EoT. *EoT* end of treatment, *MoCA* Montreal Cognitive Assessment

**Fig. 3:**
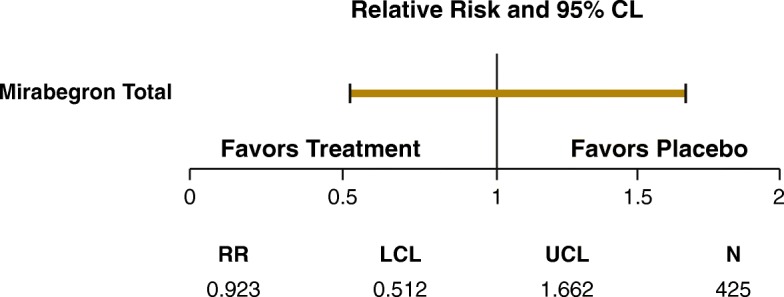
Forest plot for MCD of at least −4 points in total MoCA score. *CL* confidence limit, *EoT* end of treatment, *LCL* lower confidence limit, *MCD* minimally clinically important difference, *MoCA* Montreal Cognitive Assessment, *N* number of observations, *RR* relative risk ratio, *UCL* upper confidence limit

There were no changes in adjusted mean (SE) MoCA total score from baseline to EoT in the mirabegron group (−0.2 [0.1]) or the placebo group (−0.1 [0.1]) (Table 3). Changes in subscale scores are shown in Table 4. The number of patients missing scores at EoT were 29 for placebo and 18 for the mirabegron total group. Of the 411 patients receiving placebo and 425 patients receiving mirabegron, 48 patients (24 in each group) had declines of MoCA score of ≥4 points at week 12. The distribution of score changes are shown in Fig. 2. At week 12/EoT, 24.5% (104/425) and 25.8% (106/411) of mirabegron and placebo patients, respectively, had impaired cognitive function (Fig. 1). Post hoc analyses showed mirabegron and placebo to be similar with respect to the likelihood of experiencing cognitive decline (Fig. 3), using the MDC for a community-living elder population found in the literature [25]. MoCA scores showed no differences in change from baseline to EoT by baseline medical history of depression or antidepressant medication use, or by presence of ≥1 strong anticholinergic concomitant medication as recognized by the Anticholinergic Cognitive Burden scale [26] (Table 5).

**Table 4:** Change from baseline to EoT in MoCA subscale scores (SAF)

Change, mean (SD)	Placebo (*n* = 411)	Mirabegron Total (*n* = 425)
Attention Points	−0.0 (0.9)	−0.1 (0.9)
Language Points	0.0 (0.7)	0.0 (0.7)
Naming Points	−0.0 (0.4)	0.0 (0.4)
Visuospatial/Executive Points	0.0 (1.0)	−0.1 (1.2)
Abstraction Points	0.0 (0.5)	−0.0 (0.5)
Delayed Recall Points	0.2 (1.4)	0.2 (1.3)
Orientation Points	−0.0 (0.4)	−0.0 (0.4)

*EoT* end of treatment, *MoCA* Montreal Cognitive Assessment, *SAF* safety analysis set, *SD* standard deviation

**Table 5:** MoCA scores for patients with/without use of antidepressant medication, depression diagnosis from medical history, and by use of strong anticholinergic burden concomitant medication

	Placebo (*n* = 411)			Mirabegron Total (*n* = 425)		
	Total score at BL, mean (SD)	Total score at EoT, mean (SD)	Change in Total score, mean	Total score at BL, mean (SD)	Total score at EoT, mean (SD)	Change in Total score, mean
Use of antidepressant medication^a^						
No (*n* = 695)	26.9 (2.9)	27.1 (2.8)	0.2	26.9 (2.7)	27.0 (2.7)	0.0
Yes (*n* = 141)	26.6 (2.6)	26.9 (2.4)	0.3	26.5 (3.0)	26.6 (2.8)	0.2
Depression diagnosis from medical history^b^						
No (*n* = 683)	26.8 (2.9)	27.0 (2.7)	0.2	27.0 (2.5)	27.0 (2.7)	0.0
Yes (*n* = 153)	26.9 (2.6)	27.1 (2.6)	0.1	26.2 (3.3)	26.7 (2.8)	0.4
Use of anticholinergic burden concomitant medication^c^						
No (*n* = 767)	26.8 (2.8)	27.1 (2.7)	0.2	26.8 (2.7)	26.9 (2.7)	0.0
Yes (*n* = 69)	26.6 (3.5)	26.6 (3.1)	0	27.0 (2.9)	27.4 (2.4)	0.4

^a^*N* = 345 for placebo and 350 for mirabegron total for No, *N* = 66 for placebo and 75 for mirabegron total for Yes

^b^*N* = 341 for placebo and 342 for mirabegron total for No, *N* = 70 for placebo and 83 for mirabegron total for Yes

^c^*N* = 380 for placebo and 387 for mirabegron total for No, *N* = 31 for placebo and 38 for mirabegron total for Yes

Of note, over 300 study participants, or nearly 41%, experienced an increase from one to nine points in MoCA score (Fig. 2). There were no differences between treatment and placebo groups in MoCA score improvements. The frequency of AEs that are typically seen with antimuscarinics was low for both mirabegron and placebo (Table 6).

**Table 6:** Drug-related treatment-emergent adverse events ≥1% in either treatment group (SAF)

TEAEs, *n* (%)	Placebo (*n* = 442)	Mirabegron Total (*n* = 445)
Drug-related TEAEs^a^	57 (12.9)	84 (18.9)
Dry mouth	7 (1.6)	6 (1.3)
Nausea	3 (0.7)	6 (1.3)
Constipation	4 (0.9)	3 (0.7)
Diarrhea	1 (0.2)	5 (1.1)
Headache	7 (1.6)	18 (4.0)
Dizziness	6 (1.4)	2 (0.4)
Somnolence	1 (0.2)	2 (0.4)
Escherichia urinary tract infection	7 (1.6)	9 (2.0)
Fatigue	10 (2.3)	7 (1.6)

^a^Possible or probable, as assessed by the investigator, or where relationship was missing

*SAF* safety analysis set, *TEAEs* treatment-emergent adverse events

## Discussion

Treatment with mirabegron for 12 weeks had no adverse impact on cognitive function in patients aged ≥65 years, as measured by the MoCA and compared with those randomized to placebo. Together with the efficacy demonstrated during the PILLAR study, these data suggest that mirabegron does not worsen cognition in older adults treated for OAB who are at risk of or concerned about cognitive impairment. Therefore, mirabegron represents a viable alternative for treatment of older patients with OAB, especially for those on anticholinergic medications for other diseases.

In comparison, a systematic review and several subsequent publications have found a strong link between cognitive impairment and anticholinergic potency of medications [14, 15, 26, 27]. There appears to be a differential effect on cognition between antimuscarinics used in the treatment of OAB, although there is substantial variability in scientific approaches such as duration of observation, outcome measures employed, and populations studied. Several studies have reported that oral oxybutynin has a significant negative effect on cognitive function in healthy adults ≥60 years of age [28–31]. Cognitive impairment has also been reported with tolterodine at therapeutic doses in small numbers of cognitively intact [32–34] and cognitively impaired older people [35]. In separate studies, cumulative exposure to strong anticholinergics increased the odds of transitioning from normal cognition to MCI in adults ≥65 years [36] and was associated with an increased risk for dementia and Alzheimer’s disease [15]. Anticholinergics have also been suggested to reduce the likelihood of reverting to normal cognition among those with a diagnosis of MCI [36]. However, conflicting evidence in older people with dementia suggests that anticholinergics may not affect cognition in these individuals [27, 37, 38].

It is worth noting that oxybutynin and tolterodine made up the majority of OAB medication use in these prior reports. Other studies support the lack of cognitive effects for trospium in patients ≥75 years [39–42]. Solifenacin at a dose of 10 mg also had no detectable effect on cognition in healthy elderly volunteers ≥65 years [29] or in those ≥75 years with MCI [31]. With the exception of a case report of delirium following fesoterodine treatment in an 89-year-old man with renal failure [43], there do not appear to be reports of cognitive impairment with fesoterodine in patients ≥65 years [44, 45], or with darifenacin in patients ≥60 years [30] or ≥65 years [46]. Newer, longer-acting antimuscarinics may therefore not be associated with cognitive effects; however, caution is warranted because of overall anticholinergic load.

As people aged ≥65 years are frequently under-represented in clinical trials, the results in the PILLAR study, which included a substantial proportion ≥75 years, are clinically relevant. Patients in this study were community dwelling and relatively healthy, as shown by low baseline Charlson Comorbidity Index scores. However, the number of concomitant medications and comorbid conditions observed at baseline in this study are similar to those in the general older population [8].

The MoCA presents several advantages compared with the MMSE. For example, it includes more executive function and visuospatial items than the MMSE, thus the MoCA provides a more comprehensive assessment across cognitive domains. In addition, the MoCA 5-word recall task is better at probing for subtle memory changes than the MMSE 3-word task [47]. The improvement in sensitivity in detecting cognitive decline compared with the MMSE has been clearly demonstrated, particularly in stroke studies [48, 49]. Other advantages of the MoCA are that it can be administered in the clinical setting by a trained rater in 10 min (although training occurred for all sites it is worth noting that this study took place before mandatory MoCA training was introduced by the developers), without relying on specific expertise in neurology, psychiatry, or clinical psychology. The MoCA may therefore be useful to practitioners if they are concerned about cognitive decline in their patients due to an increased anticholinergic burden resulting from multiple medications [50].

A number of studies have assessed the ability of the MoCA to detect change in healthy and impaired populations. Feeney et al. provided a distribution-based estimate of change from a sample of community-living people aged ≥55 years in Ireland [25]. This study provided a calculation of the MDC, with the implication that an individual’s score would need to change by ≥4 points on the MoCA to be confident that the change was not due to chance or measurement error. In the current study, post hoc analyses utilizing the MDC showed that mirabegron and placebo were similar with respect to the likelihood of experiencing cognitive decline.

Although initially developed for capture of MCI in patients presenting with cognitive complaints due to Alzheimer’s disease [19], the MoCA has also been used to assess cognitive decline associated with other conditions. These include stroke [51], acquired brain injury [52], Parkinson’s disease [53, 54], chronic obstructive pulmonary disease [55], heart failure [56], complications of diabetes [57], chronic hemodialysis [58], and Huntington’s disease [59]. The Huntington Study Group used the MoCA in a trial of dutetrabenazine for treatment of chorea in patients with Huntington’s disease. They reported that there was no significant difference in the mean change on the total MoCA score between placebo and the treatment of interest across the 12 weeks of the trial [59]. This outcome reflects the use of the MoCA in the current study.

The MoCA was developed specifically for detecting decrements in the performance of basic neurocognitive tasks, although it has also been used to track improvements. The current study supports the use of the MoCA for measuring decline in cognitive function as a side effect of OAB treatment and also demonstrates the similarity in general cognitive function outcomes between mirabegron and placebo. While the MoCA has well-established utility as a screening tool for cognitive impairment, it is not a diagnostic test and provides incomplete measurement of the individual core cognitive domains. It should therefore not be viewed as a substitute for more in-depth neuropsychological assessment when domain-specific information is required [60].

Limitations of the current study include the short study duration and the use of the same version of the MoCA 12 weeks after first administration. This use of the same version is likely reflected in the 41% of patients who improved their MoCA score on subsequent administration (i.e. a training effect). In addition, the study enrolled patients with wet OAB based in the community, so the results are not representative of other elderly patient groups, such as those in nursing homes or hospitalized. Also, Charlson Comorbidity Index scores were very low at baseline, indicating that these community-dwelling patients were generally healthy and unlikely to be frail; thus these findings do not apply to all elderly individuals with OAB.

## Conclusions

Cognitive assessment over 12 weeks using the MoCA in the PILLAR study demonstrates that mirabegron treatment does not contribute to drug-related cognitive side-effects. Furthermore, the pattern of change in cognition over the trial duration in an older OAB trial population does not appear to differ from that of subjects receiving placebo.

## Data Availability

Access to anonymized individual participant-level data collected during the trial, in addition to supporting clinical documentation, is planned for trials conducted with approved product indications and formulations, as well as compounds terminated during development. Conditions and exceptions are described under the Sponsor Specific Details for Astellas on www.clinicalstudydatarequest.com. Study-related supporting documentation is redacted and provided if available, such as the protocol and amendments, statistical analysis plan and clinical study report. Access to participant-level data is offered to researchers after publication of the primary manuscript (if applicable) and is available as long as Astellas has legal authority to provide the data. Researchers must submit a proposal to conduct a scientifically relevant analysis of the study data. The research proposal is reviewed by an Independent Research Panel. If the proposal is approved, access to the study data is provided in a secure data sharing environment after receipt of a signed Data Sharing Agreement.

## Abbreviations

AEs: Adverse events
BBB: Blood-brain barrier
EoT: End of treatment
LUTS: Lower urinary tract symptoms
MCI: Mild cognitive impairment
MDC: Minimum detectable change
MMSE: Mini-Mental State Examination
MoCA: Montreal Cognitive Assessment
NOBLE: National Overactive Bladder Evaluation
OAB: Overactive bladder

## References

[1] Milsom I, Stewart W, Thüroff J (2000). The prevalence of overactive bladder. Am J Manag Care.

[2] Stewart WF, Van Rooyen JB, Cundiff GW, Abrams P, Herzog AR, Corey R (2003). Prevalence and burden of overactive bladder in the United States. World J Urol.

[3] Coyne KS, Sexton CC, Thompson CL, Milsom I, Irwin D, Kopp ZS (2009). The prevalence of lower urinary tract symptoms (LUTS) in the USA, the UK and Sweden: results from the Epidemiology of LUTS (EpiLUTS) study. BJU Int.

[4] Irwin DE, Milsom I, Hunskaar S, Reilly K, Kopp Z, Herschorn S (2006). Population-based survey of urinary incontinence, overactive bladder, and other lower urinary tract symptoms in five countries: results of the EPIC study. Eur Urol.

[5] Litman HJ, McKinlay JB (2007). The future magnitude of urological symptoms in the USA: projections using the Boston Area Community Health survey. BJU Int.

[6] Zarowitz BJ, Allen C, O'Shea T, Tangalos E, Berner T, Ouslander JG (2015). Clinical burden and nonpharmacologic management of nursing facility residents with overactive bladder and/or urinary incontinence. Consult Pharm.

[7] Soliman Y, Meyer R, Baum N (2016). Falls in the elderly secondary to urinary symptoms. Rev Urol.

[8] Ganz ML, Liu J, Zou KH, Bhagnani T, Luo X (2016). Real-world characteristics of elderly patients with overactive bladder in the United States. Curr Med Res Opin.

[9] Wolff GF, Kuchel GA, Smith PP (2014). Overactive bladder in the vulnerable elderly. Res Rep Urol.

[10] Kistler KD, Xu Y, Zou KH, Ntanios F, Chapman DS, Luo X (2018). Systematic literature review of clinical trials evaluating pharmacotherapy for overactive bladder in elderly patients: an assessment of trial quality. Neurourol Urodyn.

[11] DuBeau Catherine E., Kuchel George A., Johnson II Theodore, Palmer Mary H., Wagg Adrian (2010). Incontinence in the frail elderly: Report from the 4th international consultation on incontinence. Neurourology and Urodynamics.

[12] Fortin M-P, Rouch I, Dauphinot V, Gédéon C, Genthon S, Bonnefoy M (2011). Effects of anticholinergic drugs on verbal episodic memory function in the elderly: a retrospective, cross-sectional study. Drugs Aging.

[13] Ancelin ML, Artero S, Portet F, Dupuy A-M, Touchon J, Ritchie K (2006). Non-degenerative mild cognitive impairment in elderly people and use of anticholinergic drugs: longitudinal cohort study. BMJ..

[14] Campbell N, Boustani M, Limbil T, Ott C, Fox C, Maidment I (2009). The cognitive impact of anticholinergics: A clinical review. Clin Interv Aging.

[15] Gray SL, Anderson ML, Dublin S, Hanlon JT, Hubbard R, Walker R (2015). Cumulative use of strong anticholinergics and incident dementia: a prospective cohort study. JAMA Intern Med.

[16] By the American Geriatrics Society 2015 Beers Criteria Update Expert Panel. American Geriatrics Society 2015 updated Beers Criteria for potentially inappropriate medication use in older adults. J Am Geriatr Soc. 2015;63(11):2227–46. 10.1111/jgs.13702 26446832

[17] Folstein MF, Folstein SE, McHugh PR (1975). "Mini-mental state": A practical method for grading the cognitive state of patients for the clinician. J Psychiatr Res.

[18] Copeland JRM, Dewey ME, Henderson AS, Kay DWK, Neal CD, Harrison MAM (1988). The Geriatric Mental State (GMS) used in the community: replication studies of the computerized diagnosis AGECAT. Psychol Med.

[19] Nasreddine ZS, Phillips NA, Bédirian V, Charbonneau S, Whitehead V, Collin I (2005). The Montreal Cognitive Assessment, MoCA: a brief screening tool for mild cognitive impairment. J Am Geriatr Soc.

[20] Drake MJ, MacDiarmid S, Chapple CR, Esen A, Athanasiou S, Cambronero Santos J (2017). Cardiovascular safety in refractory incontinent patients with overactive bladder receiving add-on mirabegron therapy to solifenacin (BESIDE). Int J Clin Pract.

[21] Yoshida M, Nozawa Y, Kato D, Tabuchi H, Kuroishi K (2019). Safety and effectiveness of mirabegron in patients with overactive bladder aged ≥75 years: analysis of a Japanese post-marketing study. Low Urin Tract Symptoms.

[22] Wagg A, Cardozo L, Nitti VW, Castro-Diaz D, Auerbach S, Blauwet MB (2014). The efficacy and tolerability of the β3-adrenoceptor agonist mirabegron for the treatment of symptoms of overactive bladder in older patients. Age Ageing.

[23] Wagg A, Nitti VW, Kelleher C, Castro-Diaz D, Siddiqui E, Berner T (2016). Oral pharmacotherapy for overactive bladder in older patients: mirabegron as a potential alternative to antimuscarinics. Curr Med Res Opin.

[24] Wagg Adrian, Staskin David, Engel Eli, Herschorn Sender, Kristy Rita M., Schermer Carol R. (2020). Efficacy, safety, and tolerability of mirabegron in patients aged ≥65 yr with overactive bladder wet: a phase IV, double-blind, randomised, placebo-controlled study (PILLAR). European Urology.

[25] Feeney J, Savva GM, O'Regan C, King-Kallimanis B, Cronin H, Kenny RA (2016). Measurement error, reliability, and minimum detectable change in the Mini-Mental State Examination, Montreal Cognitive Assessment, and Color Trails Test among community living middle-aged and older adults. J Alzheimers Dis.

[26] Boustani M, Campbell N, Munger S, Maidment I, Fox C (2008). Impact of anticholinergics on the aging brain: a review and practical application. Aging Health.

[27] Fox C, Richardson K, Maidment ID, Savva GM, Matthews FE, Smithard D (2011). Anticholinergic medication use and cognitive impairment in the older population: the Medical Research Council cognitive function and ageing study. J Am Geriatr Soc.

[28] Katz IR, Sands LP, Bilker W, DiFilippo S, Boyce A, D'Angelo K (1998). Identification of medications that cause cognitive impairment in older people: the case of oxybutynin chloride. J Am Geriatr Soc.

[29] Wesnes KA, Edgar C, Tretter RN, Bolodeoku J (2009). Exploratory pilot study assessing the risk of cognitive impairment or sedation in the elderly following single doses of solifenacin 10 mg. Expert Opin Drug Saf.

[30] Kay G, Crook T, Rekeda L, Lima R, Ebinger U, Arguinzoniz M (2006). Differential effects of the antimuscarinic agents darifenacin and oxybutynin ER on memory in older subjects. Eur Urol.

[31] Wagg A, Dale M, Tretter R, Stow B, Compion G (2013). Randomised, multicentre, placebo-controlled, double-blind crossover study investigating the effect of solifenacin and oxybutynin in elderly people with mild cognitive impairment: the SENIOR study. Eur Urol.

[32] Tsao JW, Heilman KM (2003). Transient memory impairment and hallucinations associated with tolterodine use. N Engl J Med.

[33] Salvatore S, Serati M, Cardozo L, Uccella S, Bolis P (2007). Cognitive dysfunction with tolterodine use. Am J Obstet Gynecol.

[34] Womack KB, Heilman KM (2003). Tolterodine and memory: dry but forgetful. Arch Neurol.

[35] Jewart RD, Green J, Lu C-J, Cellar J, Tune LE (2005). Cognitive, behavioral, and physiological changes in Alzheimer disease patients as a function of incontinence medications. Am J Geriatr Psychiatry.

[36] Campbell NL, Lane KA, Gao S, Boustani MA, Unverzagt F (2018). Anticholinergics influence transition from normal cognition to mild cognitive impairment in older adults in primary care. Pharmacotherapy..

[37] Lackner TE, Wyman JF, McCarthy TC, Monigold M, Davey C (2008). Randomized, placebo-controlled trial of the cognitive effect, safety, and tolerability of oral extended-release oxybutynin in cognitively impaired nursing home residents with urge urinary incontinence. J Am Geriatr Soc.

[38] Sink KM, Thomas J, Xu H, Craig B, Kritchevsky S, Sands LP (2008). Dual use of bladder anticholinergics and cholinesterase inhibitors: long-term functional and cognitive outcomes. J Am Geriatr Soc.

[39] Chancellor M, Boone T (2012). Anticholinergics for overactive bladder therapy: central nervous system effects. CNS Neurosci Ther.

[40] Staskin D, Kay G, Tannenbaum C, Goldman HB, Bhashi K, Ling J (2010). Trospium chloride is undetectable in the older human central nervous system. J Am Geriatr Soc.

[41] Geller EJ, Dumond JB, Bowling JM, Khandelwal CM, Wu JM, Busby-Whitehead J (2017). Effect of trospium chloride on cognitive function in women aged 50 and older: a randomized trial. Female Pelvic Med Reconstr Surg.

[42] Staskin D, Kay G, Tannenbaum C, Goldman HB, Bhashi K, Ling J (2010). Trospium chloride has no effect on memory testing and is assay undetectable in the central nervous system of older patients with overactive bladder. Int J Clin Pract.

[43] Charbonneau JM, Bisset R, Nguyen PV-Q (2016). Delirium following fesoterodine treatment for urgency incontinence in an 89-year old man. Can Urol Assoc J.

[44] DuBeau CE, Kraus SR, Griebling TL, Newman DK, Wyman JF, Johnson TM (2014). Effect of fesoterodine in vulnerable elderly subjects with urgency incontinence: a double-blind, placebo controlled trial. J Urol.

[45] Kay GG, Maruff P, Scholfield D, Malhotra B, Whelan L, Darekar A (2012). Evaluation of cognitive function in healthy older subjects treated with fesoterodine. Postgrad Med.

[46] Lipton RB, Kolodner K, Wesnes K (2005). Assessment of cognitive function of the elderly population: effects of darifenacin. J Urol.

[47] Lam B, Middleton LE, Masellis M, Stuss DT, Harry RD, Kiss A (2013). Criterion and convergent validity of the Montreal cognitive assessment with screening and standardized neuropsychological testing. J Am Geriatr Soc.

[48] Koski L (2013). Validity and applications of the Montreal cognitive assessment for the assessment of vascular cognitive impairment. Cerebrovasc Dis.

[49] Lees R, Selvarajah J, Fenton C, Pendlebury ST, Langhorne P, Stott DJ (2014). Test accuracy of cognitive screening tests for diagnosis of dementia and multidomain cognitive impairment in stroke. Stroke..

[50] Yoshida M, Kato D, Nishimura T, Van Schyndle J, Uno S, Kimura T (2018). Anticholinergic burden in the Japanese elderly population: Use of antimuscarinic medications for overactive bladder patients. Int J Urol.

[51] Burton L, Tyson SF (2015). Screening for cognitive impairment after stroke: a systematic review of psychometric properties and clinical utility. J Rehabil Med.

[52] Lim PA, McLean AM, Kilpatrick C, DeForge D, Iverson GL, Silverberg ND (2016). Temporal stability and responsiveness of the Montreal Cognitive Assessment following acquired brain injury. Brain Inj.

[53] Biundo R, Weis L, Bostantjopoulou S, Stefanova E, Falup-Pecurariu C, Kramberger MG (2016). MMSE and MoCA in Parkinson's disease and dementia with Lewy bodies: a multicenter 1-year follow-up study. J Neural Transm (Vienna).

[54] Fiorenzato E, Weis L, Falup-Pecurariu C, Diaconu S, Siri C, Reali E (2016). Montreal Cognitive Assessment (MoCA) and Mini-Mental State Examination (MMSE) performance in progressive supranuclear palsy and multiple system atrophy. J Neural Transm (Vienna).

[55] Villeneuve S, Pepin V, Rahayel S, Bertrand J-A, de Lorimier M, Rizk A (2012). Mild cognitive impairment in moderate to severe COPD: a preliminary study. Chest..

[56] Cameron J, Worrall-Carter L, Page K, Baker SS, Ski CF (2013). Screening for mild cognitive impairment in patients with heart failure: Montreal Cognitive Assessment versus Mini Mental State Exam. Eur J Cardiovasc Nurs.

[57] Oğurel T, Oğurel R, Özer MA, Türkel Y, Dağ E, Örnek K (2015). Mini-mental state exam versus Montreal Cognitive Assessment in patients with diabetic retinopathy. Niger J Clin Pract.

[58] Tiffin-Richards FE, Costa AS, Holschbach B, Frank RD, Vassiliadou A, Krüger T (2014). The Montreal Cognitive Assessment (MoCA) - a sensitive screening instrument for detecting cognitive impairment in chronic hemodialysis patients. PLoS One.

[59] Huntington Study Group. Effect of deutetrabenazine on chorea among patients with Huntington disease: a randomized clinical trial. JAMA. 2016;316(1):40–50. 10.1001/jama.2016.8655 27380342

[60] Coen RF, Robertson DA, Kenny RA, King-Kallimanis BL (2016). Strengths and limitations of the MoCA for assessing cognitive functioning: findings from a large representative sample of Irish older adults. J Geriatr Psychiatry Neurol.

